# Reduced antioxidant defense in early onset first-episode psychosis: a case-control study

**DOI:** 10.1186/1471-244X-11-26

**Published:** 2011-02-14

**Authors:** Juan Antonio Micó, Maria Olga Rojas-Corrales, Juan Gibert-Rahola, Mara Parellada, Dolores Moreno, David Fraguas, Montserrat Graell, Javier Gil, Jon Irazusta, Josefina Castro-Fornieles, Cesar Soutullo, Celso Arango, Soraya Otero, Ana Navarro, Inmaculada Baeza, Mónica Martínez-Cengotitabengoa, Ana González-Pinto

**Affiliations:** 1 Department of Neuroscience, Pharmacology and Psychiatry, School of Medicine, CIBERSAM, Centro de Investigación Biomédica en Red de Salud Mental. University of Cádiz, Spain; 2 Adolescent Unit, Department of Psychiatry, Hospital General Universitario Gregorio Marañón, CIBERSAM, Centro de Investigación Biomédica en Red de Salud Mental Madrid, Spain; 3 Mental Health Service. University Hospital, Albacete. Centro de Investigación Biomédica en Red de Salud Mental, CIBERSAM, Spain; 4 Section of Child and Adolescent Psychiatry and Psychology, Hospital Infantil Universitario Niño Jesús, Madrid, Spain; 5 Department of Physiology, Faculty of Medicine and Dentistry, University of the Basque Country, Bilbao, Bizkaia, Spain; 6 Department of Child and Adolescent Psychiatry and Psychology, Institut Clinic of Neurosciences, IDIBAPS, (Institut d'Investigacions Biomèdiques August Pi Sunyer), Hospital Clínic Universitari of Barcelona, CIBERSAM, Centro de Investigación Biomédica en Red de Salud Mental, Spain; 7 Child & Adolescent Psychiatry Unit, Department of Psychiatry & Medical Psychology, University of Navarra, Pamplona, Spain; 8 Child And Adolescent Psychiatry Unit, Department of Psychiatry, Valdecilla Universiy Hospital, Santander, Cantabria, Spain; 9 Department of Biochemistry and Molecular Biology, School of Medicine, University of Cádiz, CIBERSAM, Centro de Investigación Biomédica en Red de Salud Mental, Spain; 10 Hospital Santiago, Department of Psychiatry, CIBERSAM, Centro de Investigación Biomédica en Red de Salud Mental, University of the Basque Country, Olaguibel 29, Vitoria, Spain

## Abstract

**Background:**

Our objective is to determine the activity of the antioxidant defense system at admission in patients with early onset first psychotic episodes compared with a control group.

**Methods:**

Total antioxidant status (TAS) and lipid peroxidation (LOOH) were determined in plasma. Enzyme activities and total glutathione levels were determined in erythrocytes in 102 children and adolescents with a first psychotic episode and 98 healthy controls.

**Results:**

A decrease in antioxidant defense was found in patients, measured as decreased TAS and glutathione levels. Lipid damage (LOOH) and glutathione peroxidase activity was higher in patients than controls. Our study shows a decrease in the antioxidant defense system in early onset first episode psychotic patients.

**Conclusions:**

Glutathione deficit seems to be implicated in psychosis, and may be an important indirect biomarker of oxidative stress in early-onset schizophrenia. Oxidative damage is present in these patients, and may contribute to its pathophysiology.

## Background

Oxidative stress-induced impairment of neuronal processes has been reported to be involved in neurodegeneration [1] and also in the pathophysiology of neuropsychiatric diseases such as schizophrenia [2-6]. It represents an imbalance between the oxidant molecules and the antioxidant defense system, and generally occurs as a consequence of increased production of reactive oxygen species (ROS), or when the antioxidant defense system is inefficient.

The primary antioxidant cellular defense is enzymatic involving superoxide dismutase (SOD), catalase (CAT) and glutathione peroxidase (cGPx), which are constitutively expressed in all tissues. Glutathione (GSH) is the main non-enzymatic cellular antioxidant, regulated by two enzymes, glutathione peroxidase and glutathione reductase, which play a critical role in protecting cells from damage by ROS generated by dopamine metabolism. However, there are conflicting data in the literature on the activities or levels of antioxidant enzymes in bipolar disorder [7] and schizophrenia. SOD activity in erythrocytes of schizophrenia patients has been reported to be increased [8-11], decreased [12-16] or unchanged [17-19]. cGPx activities have been reported to be unchanged [6,10,12,14,18] but also increased [8,20] or decreased [15,21], and CAT activity has been found unchanged [14,18,19], increased [13,22] and decreased [10,23]. SOD levels have been reported to be increased in plasma [24] and in brain tissues [9]. Some authors suggest the importance of measuring the total antioxidant status (TAS) in some mental diseases [21-23]. The early studies of Golse et al. [24-26] founded altered the levels of SOD and cGPx in children with early-onset psychosis.

To our knowledge there are few published studies that have evaluated the oxidative balance in psychosis with an early age of onset, which has been associated with a more severe form of the disease than the adult-onset form. Thus, child- and adolescent-onset first episode psychosis needs to be further studied in order to identify its specific features, differential diagnosis, treatment of choice and outcome [27,28].

Our goal is to assess oxidative stress at the peripheral level using measures that could serve as an index of the pathophysiological changes occurring in the brain of patients with early-onset first psychotic episodes of short duration. We hypothesize that patients with early psychosis will have increased oxidative stress in comparison with a healthy control group.

## Methods

### Subjects

The Child and Adolescent First-Episode Psychosis Study (CAFEPS) was a case-control study that included 110 patients with first-episode psychosis (FEP) and a history of less than 6 months of psychotic symptoms, and 98 healthy controls. Of this sample, 102 patients and 95 controls had blood samples available for determining antioxidant status and were included in the present study. Diagnosis was confirmed according to the DSM-IV criteria [29], using the Kiddie Schedule for Affective Disorders and Schizophrenia, Present and Lifetime version (K-SADS-PL) [30], Spanish translation [31] at admission and according to the DSM-IV criteria at one year follow-up. In the analyses, the problem of diagnostic instability was avoided by grouping the patients according to their confirmed diagnosis at one-year follow-up. The study design and recruitment, and the clinical and demographic characteristics of the sample have been described elsewhere [32]. Clinical symptoms were assessed using the following scales: the Positive and Negative Symptoms Scale (PANSS), the Hamilton Depression Rating Scale (HDRS)-21, the Young Mania Rating Scale (YMRS) and the Global Assessment Functioning (GAF). Neither the patients nor the control group had participated in heavy exercise or had fasted during the week before study entry. The study was approved by the Ethics and Clinical Research Boards of all the Hospitals involved in the study. Parents or legal representatives gave written informed consent and participants assented to participate in the study.

## Methods

Blood samples were collected in heparin-containing tubes immediately after enrolment and processed following standard procedures. Blood cells and plasma were immediately frozen and stored at -80°C until analysis. All samples were analyzed in a single batch. Oxidative stress was evaluated by measuring primary enzymatic antioxidant defense (cellular glutathione peroxidase, catalase and superoxide dismutase activities) and plasma levels of total antioxidants, glutathione and lipid peroxidation. Total antioxidant status (TAS) and lipid peroxidation were determined by standardized spectrophotometric assays (Bioxytech) in plasma. Briefly, the TAS assay [33] relies on the ability of antioxidants present in plasma to inhibit the oxidation of ABTS (2,2'-azino-bis(3-ethylbenzthiazoline-6-sulphonicacid)), which is monitored by reading the absorbance at 600 nm. Lipid peroxidation was measured using an assay for lipid hydroperoxides (LOOHs), which is based on the oxidation of ferrous ions to ferric ions by hydroperoxides under acidic conditions [34]. Enzyme activities and total GSH levels were also determined by standardized spectrophotometric assays in haemolysates of erythrocytes. Total GSH was measured using the Bioxytech GSH-420 assay kit, which uses a method based on the formation of a chromophoric thione by specific elimination of GSH-thioether; the absorbance measured at 420 nm is directly proportional to the total GSH concentration. The assay for measuring CAT activity involved a two step process: (1) a sample containing catalase was incubated in the presence of a known concentration of hydrogen perioxide (H_2_O_2_); (2) the remaining hydrogen peroxide in the reaction mixture facilitated an oxidative coupling reaction and the resulting quinoneimine dye (which correlates to the amount of hydrogen peroxide) was measured at 520 nm [35]. cGPx enzyme activity was determined using the method described by Paglia and Valentine in 1967 [36], where the oxidation of NADPH to NADP+ was monitored by a decrease in absorbance at 340 nm. SOD activity determination [37] was based on the SOD-mediated increase in the rate of auto-oxidation of a tetracyclic catechol in an aqueous alkaline solution to yield a chromophore with maximum absorbance at 525 nm. All the assays were performed in a diode-array detector coupled to a thermostatically-controlled water bath.

### Statistical analyses

Statistical analyses were carried out with SPSS v14.0 (Statistical Package for the Social Sciences, Chicago, USA). For descriptive purposes, the continuous variables were expressed as means ± standard deviation (SD); ranges and percentages were used to describe the categorical variables.

To perform the bivariate analyses, Mann-Whitney U tests were used for continuous variables and contingency tables for categorical variables (χ^2 ^or Fisher's exact test if n≤5 per cell). To assess age differences between diagnostic groups, Kruskal-Wallis H tests were used. The associations between continuous variables were calculated using nonparametric (Spearman) correlations. Significance was defined as two-tailed p < 0.05 at stated degrees-of-freedom (df).

## Results

### Sample characteristics

Age and gender distributions of the subjects did not differ between patients and controls or between the different diagnostic subgroups of patients (Table 1). To avoid diagnostic instability, the diagnostic spectra were confirmed after one year of follow-up and the patients were grouped as follows: schizophrenia spectrum disorders (SCH) (48.04%), which includes schizophrenia, schizophreniform and schizoaffective disorders; psychotic disorders not otherwise specified (PNOS) (24.51%); psychotic bipolar disorder (BIP) (17.65%); and depressive disorder with psychotic symptoms (DEP) (9.80%). When the blood samples were collected at admission, 42 patients had been receiving treatment with antipsychotic medication for some days: 37 patients took atypical antipsychotics and the other 5 took typical antipsychotics. Comparison of the oxidative stress variables and LOOH levels between the treated and drug-free patient groups showed no significant differences (TAS: U = 1303.500, p = 0.814; LOOH: U = 653.500, p = 0.978; GSH: U = 1138.000, p = 0.136; cGPx: U = 932.000, p = 0.669; CAT: U = 1080.000, p = 0.429; SOD: U = 1169.500, p = 0.127).

**Table 1:** Distribution of controls and patients by age and gender

	Controls	Patients	PNOS-_a_	SCH_b_	DEP_c_	BIP_d_
***N***	95	102	25	49	10	18

**Age (years)**						

Mean + SD	15.25 + 0.22	15.61 + 0.26	15.53 + 0.44	15.42 + 0.39	15.82 + 0.45	16.07 + 0.45

Median	16 (9-17)	16 (9-17)	16 (11-17)	16 (9-17)	16.5 (14-17)	16.5 (11-17)

Statistic	4224.51 (U Mann-Whitney)			4.57 (Kruskal-Wallis)		

p-value	0.109			0.335		

**Gender (male)**						

Frequency	61 (64.2%)	70 (66.7%)	14 (56%)	38 (77.6%)	7 (70%)	11 (61.1%)

Statistic	0.43		Fisher			

p-value	0.548		0.350			

_a_PNOS: Psychotic disorder not otherwise specified, _b_SCH: Schizophrenia spectrum disorder, _c_DEP: Depressive disorder with psychotic symptoms, _d_BIP: Psychotic bipolar disorder.

### Oxidative stress variables of patients versus control subjects

There were no sex differences between patients and controls for any of the oxidative stress parameters assessed (data not shown). When the whole sample was considered together, there were no significant associations between any of the oxidative stress variables and tobacco use, with subjects being classified as smokers if they reported using tobacco on a daily basis. However, there was a significant association between age and total antioxidant status (Spearman Rho = -0.252, p < 0.001).

Analyses of the oxidative stress variables revealed a decrease in antioxidant defense in FEP patients versus the control group, measured as decreased TAS in plasma and decreased GSH levels in erythrocyte lysates of the blood samples taken at admission. In addition, lipid damage measured as LOOHs (μM) was significantly higher in patients at admission than in control subjects. Regarding enzyme activities, only cGPx activity (U/mL) was higher in patients than in controls, with the activity of CAT and SOD being similar in both groups of subjects (Table 2).

**Table 2:** Oxidative stress variables for the control group and patients in blood samples taken at admission

Variables in blood samples ***Control N = ***94 ***Patient N = ***102	Mean	SD	U Mann-Whitney	p-value
**Total Antioxidant Status *(mM)***			2787.54	**<0.001**

*Control (N = 94)*	1.26	0.05		

*Patient (N = 102)*	0.95	0.35		

**Glutathione *(μM)***			4194	**0.001**

*Control (N = 93)*	394.12	15.91		

*Patient (N = 90)*	324.38	13.54		

**Lipid Hydroperoxides *(μM)***			2369.53	**0.018**

*Control (N = 82)*	6.17	0.47		

*Patient (N = 74)*	9.15	1.20		

**Glutathione Peroxidase *(mU/mL)***			3421.56	**0.033**

*Control (N = 93)*	913.06	59.98		

*Patient (N = 90)*	1028.91	49.55		

**Catalase *(U/mL)***			3783.54	0.262

*Control (N = 94)*	10708.92	4715.01		

*Patient (N = 110)*	10473.27	4633.22		

**Superoxide Dismutase *(U/mL)***			3700	0.176

*Control (N = 94)*	5079.55	2938.49		

*Patient (N = 110)*	6072.4	2914.30		

To determine whether changes in the antioxidant defense system were associated with membrane damage, we examined the correlation between LOOH levels and oxidative stress variables and found a significant correlation between LOOH levels and SOD (Rho = -0.190, p = 0.018).

When the oxidative stress variables were compared between controls and patients in the different diagnostic groups (Table 3), TAS was significantly lower in all the diagnostic groups, except the DEP group. Examining between-diagnostic groups differences in the oxidative stress variables, we found a significant between-group difference for TAS, with lower levels in the SCH and BIP groups (K = 25.620, p < 0.001). None of the other variables showed a between-diagnostic group difference.

**Table 3:** Oxidative stress variables for patients in the different diagnostic groups versus the control group

	N	Mean	SD	U Mann-hitney	p	N	Mean	SD	U Mann-Whitney	p
	**TAS (mM)**					**GSH (μM)**				

Control group	94	1.26	0.05			93	394.11	15.90		
SCH_b_	49	0.91	0.04	1193.50	**< 0.001**	43	308.34	20.06	1317.50	**0.005**
PNOS-_a_	25	0.99	0.09	622	**0.002**	21	327.62	29.21	555	0.195
BIP_d_	18	0.88	0.06	290.50	**0.001**	17	347.44	24.15	530.50	0.606
DEP_c_	10	1.16	0.14	232.50	0.501	9	350.27	45.23	245.50	0.841
	**LOOH (μM)**					**cGPX (mU/mL)**				
Control group	82	6.17	0.47			93	912.96	59.93		
SCH_b_	33	10.06	2.50	1431	0.161	43	1068.37	61.63	2283.50	**0.015**
PNOS-_a_	17	6.03	0.87	641	0.856	21	620.73	135.46	823.50	0.734
BIP_d_	15	10.88	1.58	713.50	**0.004**	17	1142.08	112.88	831	**0.031**
DEP_c_	9	8.82	1.60	328.50	0.083	9	728.812	80.62	228	0.447

_a_PNOS: Psychotic disorder not otherwise specified, _b_SCH: Schizophrenia spectrum disorder, _c_DEP: Depressive disorder with psychotic symptoms, _d_BIP: Psychotic bipolar disorder.

However, as shown in Table 3, GSH levels were lower only in SCH patients than in controls and lipid hydroperoxides (LOOH levels) were significantly higher only in the BIP group compared with the control group.

For enzyme activities, cGPx activity was significantly enhanced in the SCH and BIP groups. For CAT and SOD, there were no significant differences between patients and the control group or among the different diagnostic groups.

We found no association between any of the oxidative stress variables and the following clinical symptoms: positive symptoms (PANSS positive), negative symptoms (PANSS negative), and depressive symptoms. There was a negative correlation between manic symptoms (YMRS) and TAS (r = -0.255), and a positive correlation between global functioning and TAS (r = 0.235).

## Discussion

The antioxidant defense system seems to be decreased in our population of children and adolescents with a first early onset psychotic episode and who were either treatment-naïve or had been treated with antipsychotics for only a short time when the blood samples were taken at admission. In particular, we found a decreased total antioxidant status and lower GSH levels in patients compared to healthy controls. Furthermore, compared with the control group, TAS was significantly lower in each of the three main diagnostic groups (psychotic disorder not otherwise specified, bipolar disorder and schizophrenia), but GSH was significantly lower only in the schizophrenia group. Moreover, for the oxidative stress variables measured, a between-diagnostic group difference was significant only for TAS, with the lowest TAS levels in the schizophrenic and bipolar patients. From the baseline data it appears that patients with more severe and chronic diseases (SCH and BIP) have more oxidative stress than those with acute psychosis. Indeed, it must be taken into account that although the oxidative data shown were obtained at baseline, the diagnoses were only confirmed during the follow-up. These preliminary findings suggest that antioxidant problems may be a function of diagnosis, in this case schizophrenia and bipolar disorder.

Lipid oxidative damage seems to be increased in early first psychotic episode patients, measured as elevated levels of lipid hydroperoxides (LOOHs); the activity of cGPx is also elevated in patients compared with healthy controls. When comparing the patient subgroups by confirmed diagnoses with the control group, only bipolar patients had elevated LOOHs.

There is no bias in the results due to treatment use, because there were no differences in oxidative stress when we compared the patients who were treatment-naïve with those who had received antipsychotic treatment before admission. Previously, it has been stated that psychopharmacological treatment can partially reverse oxidative stress [38]. However, in our patient sample, treatment was used for only a short period before hospitalization, and we have not yet performed a longitudinal prospective analysis of the sample. We found a relationship between age and oxidative stress when the patients and controls were considered together. However, we cannot conclude that the older the age, the higher oxidative stress, as our older patients were adolescents. Other studies have not found any age-related differences in total antioxidant status in healthy volunteers [39].

A defect in antioxidant protection seems to be a common feature of patients with a first psychotic episode, but has been little explored so far in early onset psychosis and patients who have been psychotic for only a short time. This defect should be taken into account when developing neuroprotective treatments.

Despite the conflicting data found previously [40-43], abnormalities in antioxidant enzyme activities are frequently found in patients diagnosed with psychosis or bipolar disorder. This imbalance in enzyme activities reduces the efficacy of the cellular antioxidant defense, which requires a proper balance of antioxidant enzymes, and contributes to brain pathology [44]. Indeed, we found a significant correlation between SOD and LOOH levels indicating that oxidative stress is probably producing lipid damage. It has been taken into account that the brain contains almost no catalase and less cGPx than other tissues. Although these enzymes may serve as peripheral indicators of oxidative stress, this makes our results for GSH levels and SOD activity more interesting.

The GSH deficit found in this study and in previous reports [15,40,45-47] may be involved in membrane peroxidation and microlesions related to dopamine, which seem to be increased in psychosis, and suggest that GSH may be a possible indirect indicator of damage in neuronal membranes [48-52]. Anomalies in GSH metabolism were also supported by the low expression of the gene of the key GSH-synthesizing enzyme, glutamate cysteine ligase modifier subunit, in patient fibroblasts [53]. Moreover, inhibition of brain glutathione synthesis and dopamine uptake in developing rats induces long-term cognitive deficits in adulthood [54]. Therefore, it seems that dopamine, which has a neurotoxic potential, contributes to cellular oxidative stress, which can be exacerbated if glutathione synthesis is compromised [55]. In addition, the concentrations of glutathione and one of its metabolites, glutamylglutamine, are reduced in the cerebrospinal fluid of drug-free patients [56]. An animal model of schizophrenia has recently been proposed where a redox imbalance during postnatal development induces abnormalities in cortical development [57]. Also, a recent study performed in cultured fibroblasts of patients with schizophrenia found impaired genetic and functional capacity to synthesize GSH under conditions of oxidative stress in schizophrenic patients [58]. These converging data, in agreement with our results in child and adolescent patients, indicate that psychosis is associated with an important brain glutathione deficit. One question not answered by this study is why early-onset bipolar patients (who also have dopamine metabolism alterations) do not show a significant GSH deficit. It is possible that patients diagnosed with bipolar disorder share some etiological and physiopathological mechanisms with schizophrenia, but not all. Indeed, patients with bipolar disorder usually have less cognitive dysfunction in the long term and better prognosis than patients with schizophrenia. In fact, it could be hypothesized that different etiological mechanisms converge into precipitating a psychotic episode in an adolescent, and only in individuals with a limited GSH synthesis capacity, after which the psychotic episode develops into a degenerating condition that we call schizophrenia. This could be tested by analyzing glutathione in high risk populations that are subsequently followed up.

In our patient group, the presence of manic symptoms was associated with lower antioxidant capacity. Previously, Andreazza et al. [59] demonstrated that DNA damage, probably due to oxidative stress, was increased in mania patients and was related to severity of mania. We also found that antioxidant defense was related to global functioning.

Oxidative stress has been related to DNA damage, and it has been suggested that psychopharmacological treatment can partially reverse oxidative stress. As oxidative stress could be a marker of severity in patients with medical conditions such as atherosclerosis [60] or diabetes [61,62], long-term investigations should determine whether it is also a marker of severity in patients with psychosis. It is possible that oxidative stress could also mediate the vascular damage of patients with psychosis as it has been established that patients with psychosis are at higher risk of developing cardiovascular diseases [63] and diabetes [64].

Although we should be cautious, our findings support the possibility of using peripheral markers of oxidative and antioxidative balance in patients with first-episode psychosis, taking into account the special sensitivity of the brain to oxidative damage [65].

This study has some limitations. Data are cross-sectional, diagnostic subgroups are relatively small, and it was difficult to establish in advance a sample size to perform the data analyses because of the paucity of studies with similar design characteristics; nevertheless, our patient sample is one of the largest reported to date. A second limitation is the type of recruitment centers; the majority was hospital settings with an inpatient facility and this may represent a bias towards the inclusion of more severe cases, making it difficult to generalize the results to less severe ones. Because the data presents changes in peripheral blood, further work is needed to determine if such changes adequately reflect changes in the brain and of mental state. The strengths of the study are the uniformity in age with an early onset and first episode of all psychoses, and the existence of a control group.

## Conclusions

In summary, our study shows a decrease in the antioxidant defense system in early onset first episode psychotic patients. Glutathione deficit seems to be implicated in psychosis, and may be an important indirect biomarker of oxidative stress in schizophrenia. Oxidative damage is present in these patients and, although it may not be the main cause of psychosis, it may contribute to the pathophysiology and account for a deteriorating course and poor outcome in this early onset group. Because all the patients in the study had a first early-onset psychotic episode, the data indicate ongoing oxidative injury at onset of psychosis. Moreover, because the onset of psychotic disorders during childhood and adolescence has a devastating negative impact on normal development and functioning [66], our results provide support for further study of the possible role of antioxidants as neuroprotective therapeutic strategies for schizophrenia from early stages [67]. Data from the longitudinal study will clarify the possible utility of peripheral markers of oxidative stress as prognostic factors and also the effect of antipsychotic drugs on oxidative stress.

## Abbreviations

(CAT): Catalase; (DEP): Depressive disorder with psychotic symptoms; (FEP): First-episode psychosis; (GSH): Glutathione; (cGPx): Glutathione peroxidase; (LOOH): Lipid hydroperoxides; (BIP): Psychotic bipolar disorder; (PNOS): Psychotic disorder not otherwise specified; (ROS): Reactive oxygen species; (SCH): Schizophrenia spectrum disorder; (SOD): Superoxide dismutase; (TAS): Total antioxidant status.

## Competing interests

The authors declare that they have no competing interests.

## Authors' contributions

JAM, AGP and CA designed the study and wrote the protocol.

MORC, JMGR and MP managed the literature searches.

DM, DF, MGl, JG, SO and AN contributed to the data collection.

JI, JCF and CS contributed to the analysis and interpretation of the data.

JAM wrote the manuscript.

IB and MMC helped in writing and reviewing the manuscript.

All authors have approved the final manuscript.

## Pre-publication history

The pre-publication history for this paper can be accessed here:

http://www.biomedcentral.com/1471-244X/11/26/prepub
